# Bridging the Gap: Can COVID-19 Research Help Combat African Swine Fever?

**DOI:** 10.3390/v15091925

**Published:** 2023-09-15

**Authors:** Danaya Pakotiprapha, Sakonwan Kuhaudomlarp, Ruchanok Tinikul, Sittinan Chanarat

**Affiliations:** Department of Biochemistry and Center for Excellence in Protein and Enzyme Technology, Faculty of Science, Mahidol University, Bangkok 10400, Thailand

**Keywords:** African swine fever virus, African swine fever, COVID-19, SARS-CoV-2, diagnostics, vaccines

## Abstract

African swine fever (ASF) is a highly contagious and economically devastating disease affecting domestic pigs and wild boar, caused by African swine fever virus (ASFV). Despite being harmless to humans, ASF poses significant challenges to the swine industry, due to sudden losses and trade restrictions. The ongoing COVID-19 pandemic has spurred an unparalleled global research effort, yielding remarkable advancements across scientific disciplines. In this review, we explore the potential technological spillover from COVID-19 research into ASF. Specifically, we assess the applicability of the diagnostic tools, vaccine development strategies, and biosecurity measures developed for COVID-19 for combating ASF. Additionally, we discuss the lessons learned from the pandemic in terms of surveillance systems and their implications for managing ASF. By bridging the gap between COVID-19 and ASF research, we highlight the potential for interdisciplinary collaboration and technological spillovers in the battle against ASF.

## 1. Introduction

African swine fever (ASF) is a highly contagious disease caused by African swine fever virus (ASFV), affecting both domestic pigs and wild boar. The disease is characterized by high fever, loss of appetite, weakness, vomiting, diarrhea, and respiratory difficulties [1,2,3]. Severe cases can lead to hemorrhaging, resulting in skin redness, lesions, and internal bleeding. ASFV transmission occurs through direct contact with infected pigs or contaminated fomites, such as feed, water, and equipment in pig farms [4]. Certain species of soft ticks of the *Ornithodoros* genus act as vectors and reservoirs for the virus [5]. The virus is endemic in parts of Africa, and it has also been found in some countries in Europe and Asia. Despite ASF being harmless to humans and posing no risk to food safety, its economic impact is significant, due to the necessity for culling infected pigs and the implementation of trade restrictions. Although reports of several ASF vaccines have surfaced, the absence of an effective commercial vaccine poses a major challenge to disease control efforts. Consequently, ASF inflicts devastating losses on the pig industry, necessitating urgent research to address this growing threat.

ASFV, the causative agent of ASF, is a large, enveloped double-stranded DNA virus belonging to the unique Asfarviridae family [6,7,8]. During infection, it replicates its genome inside the nucleus of the infected cells and assembles viral particles in the viral cytoplasmic factories [1,8,9,10,11]. With a genome size of approximately 170–190 kilobases, ASFV encodes over 150 putative genes, encompassing functions such as DNA replication, capsid assembly, and regulatory proteins. Its linear viral genome is enclosed within an icosahedral capsid composed of multiple subunits containing the p72 protein. Additionally, the capsid is surrounded by a plasma membrane-derived envelope that embeds several viral proteins [12,13]. ASFV exhibits remarkable stability due to its complex structure, enabling it to endure harsh environmental conditions and persist for extended periods, which complicates outbreak prevention. Despite the significant impact of ASFV, our understanding of its pathogenesis and infection mechanisms remains limited. Therefore, advancements in vaccine development, diagnostics, and therapeutics for ASF are still in their early stages, lagging behind the intricate biology of the virus. Urgent and strategic technological developments are required to effectively prevent and control the disease.

The outbreak of Coronavirus Disease 2019 (COVID-19) caused by severe acute respiratory syndrome coronavirus 2 (SARS-CoV-2) originated in Wuhan, China, in December 2019. It rapidly spread worldwide, leading to symptoms in affected individuals such as fever, cough, shortness of breath, loss of taste or smell, fatigue, and muscle aches, among others. This pandemic affected all countries, with confirmed cases in 229 countries and territories, leading to a major public health crisis [14,15]. This global pandemic has led to overwhelming burdens on healthcare systems, with over 200 million confirmed cases and up to 5 million deaths worldwide [14,16,17]. Additionally, a substantial number of individuals continue to experience post-COVID-19 conditions or long COVID. However, amidst these devastating impacts, there have been positive outcomes, including enhanced international cooperation, improved healthcare systems, and remarkable advancements in medical research and technology. This review focuses on these technological advancements derived from COVID-19 research and their potential spillover effects for preventing and controlling outbreaks of ASF. Specifically, we explore advancements in diagnostic methods, vaccines and treatments, data analysis, and disease surveillance. The lessons learned and technological progress achieved during the COVID-19 pandemic not only have the potential to aid in ASF control, but also to enhance preparedness for future outbreaks of infectious diseases.

## 2. Viral Transmission and Host Immune Responses

### 2.1. Current Status and Progress of ASFV Research

#### 2.1.1. Hosts, Transmission, and Viral Entry Mechanism of ASFV

Domestic pigs (*Sus domesticus*) and wild boar (*Sus scrofa*) are susceptible to ASFV infection, exhibiting clinical symptoms that range from hyperacute, acute, subacute, to chronic, depending on the virulence of the viral isolates [8]. In Europe, the wild boar plays a crucial role in the circulation and transmission of ASFV. Implementing control measures on their population is challenging, and this contributes to the spread of the virus to domestic pigs [18]. Although the surveillance of ASFV in wild boar populations in Asia is relatively poor, the quantitative risk of ASFV infection in wild boar has been predicted in Asian countries, with Thailand having been categorized as a country with medium risk of ASFV infection in wild boar [19]. Warthogs (*Phacochoerus africanus*) and bush pigs (*Potamchoerus larvatus*) act as natural reservoirs, allowing the virus to replicate without causing clinical symptoms or mortality in these hosts [20].

In addition to the reservoir hosts, ASFV can also be transmitted through vectors. Soft ticks belonging to the *Ornithodoros* species are considered animal vectors of ASFV, due to their ability to maintain and transmit the virus to naive wild and domestic pigs. However, tick-mediated transmission appears to be limited to African regions [5]. Within domestic pig populations, the virus spreads through direct and indirect contact with infected pigs, exposure to viral-contaminated fomites [21], and consumption of ASFV-contaminated feed [4].

Upon exposure to domestic pigs, ASFV typically enters the host’s body through the tonsil or dorsal pharyngeal region and subsequently infects primary target cells in the lymph nodes, such as monocytes, macrophages, and dendritic cells. Three viral entry models have been proposed: 1. receptor-dependent clathrin- and dynamin-mediated endocytosis [22,23], 2. actin-mediated macropinocytosis [24], and 3. Fc-receptor mediated entry [25,26].

In clathrin-mediated endocytosis, the initial viral–host interaction is facilitated by host receptors. This interaction leads to the internalization of viral particles, resulting in the formation of clathrin-coated vesicles and endosomes. Acidification of intraluminal endosome triggers viral decapsidation and fusion between the viral and endosomal membranes, thereby releasing the viral contents into the host cells. The host receptor CD163 has been suggested to play a role in viral attachment, as its expression on mature porcine macrophage correlates with ASFV infection [27]. However, CD163 was found to be non-essential for ASFV infection in porcine epithelial kidney cell lines [28], and pigs with CD163 knockdown remained susceptible to ASFV infection [29]. Therefore, CD163 may not be the sole host factor involved in viral attachment. Several ASFV proteins, including p54, have been implicated in macrophage attachment [30], and binding to dynein for viral transport within the host cell [31]. Additionally, the viral protein p30 is involved in viral internalization [30].

Unlike clathrin-mediated endocytosis, macropinocytosis is a non-selective uptake of extracellular fluid and viral particles through actin-dependent evaginations of the cell membrane, forming vacuoles [32]. This mechanism does not require specific host–virus interactions. ASFV has been observed to utilize macropinocytosis, characterized by membrane perturbations, for entry into host cells. Inhibition of the regulatory pathways of macropinocytosis has been found to significantly impact ASFV entry [24]. Once viral particles are taken up by the cell through macropinocytosis, they enter endosomes and undergo maturation, similarly to what occurs in clathrin-mediated endocytosis, as described previously.

Fc-mediated entry is another mechanism proposed for ASFV entry, which involves the attachment of sub- or non-neutralizing antibodies to viral particles, which then facilitate attachment to Fc receptors on the host cell surface. Convalescent serum from infected pigs has been shown to enhance viral infection, supporting this mechanism [25,26].

#### 2.1.2. Immune Responses to ASFV

After entering host cells, ASFV elicits both innate and adaptive responses. Similarly to other viral infections, the innate immune response relies on the recognition of pathogen-associated molecular pattern (PAMPs) by cellular pattern recognition receptors (PRRs), including Toll-like receptors (TLRs), RIG-I-like receptors (RLRs), and cytoplasmic DNA sensors. In the case of ASFV, the cytoplasmic DNA sensing involving cyclic GMP-AMP synthase (cGAS) and stimulator of interferon genes (STING) pathway is likely the primary mechanism for detecting viral DNA and inducing the release of interferons [9]. Subsequent to viral recognition, type I IFN production and NF-κB activation are promoted, leading to transcription of a multitude of genes, resulting in the synthesis of antiviral proteins and cytokines that help eliminate the virus [33,34]. Transcriptomic analysis following ASFV infection has revealed modulation in expression of several cytokines, including interferons (IFNs), interleukin (IL), and tumor necrosis factor (TNF). These cytokines are considered to play crucial roles in ASF pathogenesis and the development of excessive inflammatory responses [35].

IFNs play a critical role as the initial defense in innate immunity against viral infections. ASFV has evolved to encode numerous proteins to delay the release of IFNs. For instance, viral proteins such as pMGF505-7R, pDP96R, pI215L, and pE120R have been identified as inhibitors of the cGAS-STING signaling pathway [36,37,38]. In addition to targeting the cGAS-STING pathway, ASFV proteins also interfere with other pathways involved in IFN release. One example is the multigene family 360 member pMGF360-12L, which inhibits the nuclear translocation of NF-κB [39]. Deletion of this gene in porcine alveolar macrophages has been shown to enhance IFN I production [40]. Another viral protein, pI329L, functions as a homologue of TLRs and affects the activity of NF-κB and the expression of IFNs, likely through competitive interaction with downstream signaling molecules of TLRs [41]. Besides IFN, ASFV also encodes proteins that interfere with the production of other cytokines. For instance, pA238L has been shown to inhibit the activation of TNF-α promoter, resulting in reduced TNF-α expression [42]. L83L binds to interleukin-1β and is thought to modulate its function, although its deletion is not critical for ASFV virulence in swine [43]. In addition to cytokines, ASFV infection also modulates the level of chemokines [35]. The specific viral factors responsible for this phenomenon, however, have yet to be determined.

Apoptosis and necrosis are prominent features observed in the later stages of ASF infection within affected tissues. However, the precise molecular mechanisms governing cell death in this context remain largely elusive. Previous studies have demonstrated that the expression of ASFV proteins such as pE199L, pE183L/p54, or CD2v/EP402R in cell lines can induce apoptosis [44,45]. Apoptotic processes can be triggered during the fusion of the viral membrane with the endosomal membrane or during virus uncoating, leading to the activation of caspases [46]. Furthermore, ER stress is known to contribute to apoptosis induction [47]. Nonetheless, during the early phase of infection, ASFV actively inhibits apoptotic responses and autophagy in infected cells. The virus employs diverse anti-apoptotic mechanisms, as comprehensively reviewed in [9,48], to suppress apoptosis and enhance the production of viral progeny. Notably, specific anti-apoptotic proteins have been identified [6,49,50,51].

ASFV infection also triggers an adaptive immune response. However, it is important to note that humoral responses, which produce neutralizing antibodies upon ASFV infection or vaccination, do not provide complete protection against re-infection [52]. Recently, the focus has shifted towards understanding cellular responses during ASFV infection, which have been reviewed and might be a key to protective immunity against ASFV [53]. Identification of the key ASFV antigens that trigger host adaptive immune responses is vital for the development of effective ASF vaccines.

### 2.2. Lessons Learned from COVID-19 Research

The emergence and rapid spread of ASFV in China in 2018 resulted in a severe shortage of pork products in 2019. This shortage led to the exploration of alternative meat sources, including wildlife and exotic animals [16]. The increased consumption of these animals, coupled with the expansion of the cold-chain supply for their meat, may have created more opportunities for human contact with SARS-CoV-2, ultimately leading to its first detection in humans in 2019 [16,54]. Extensive research efforts and international collaborations have since focused on understanding the biology and pathology of SARS-CoV-2, leading to the development of vaccine and antivirals for COVID-19. In contrast, similar advancements in ASFV research have been lacking. Thus, this section aims to highlight the knowledge and technological developments in SARS-CoV-2 that could potentially be applied to ASF control and management.

SARS-CoV-2 demonstrates a broad host range, with humans currently serving as the primary host. Experimental studies have shown that SARS-CoV-2 can also infect domesticated animals, such as raccoon dogs [55], ferrets, cats [56], white-tailed deer [57], macaques [58], and minks [59]. Transmission of the virus among the same species, such as cats, ferrets, and fruit bats, is also possible [56,60], and spillover from minks to humans has been observed [59], indicating that these animals may serve as potential reservoirs for SARS-CoV-2. Moreover, cases of spillover to wild and zoo animals, including lions, Malayan tigers, and great apes, have been documented [61]. Coronaviruses closely related to SARS-CoV-2 have been found in bats [62] and pangolins [63], suggesting that these animals may serve as original or intermediate hosts.

The infection process of SARS-CoV-2 begins with the attachment of the virus to host surfaces and binding to host receptors. The susceptibility of cells and hosts to SARS-CoV-2 infection is likely influenced by the presence of primary and auxiliary receptors (as comprehensively reviewed in [64,65]). One primary receptor is angiotensin converting enzyme 2 (ACE2), which interacts with the spike (S) protein of SARS-CoV-2 [66,67]. The co-expression of ACE2 and viral antigens in various organs of postmortem specimens from COVID-19 patients, such as lungs, trachea, small intestine, kidney, and heart, suggests that ACE2 plays a crucial role in cell entry in these tissues [68]. The expression of ACE2 and host proteases reflects the tropism of SARS-CoV-2 for different cell types [68]. The ability of SARS-CoV-2 to transmit across a wide range of hosts correlates with the expression and conservation of amino acid epitopes on ACE2 orthologues that interact with the spike protein’s receptor-binding domain (RBD) [69,70].

Initially, two models were proposed for the entry of SARS-CoV-2 into human cells: 1. early entry pathway via direct fusion with the cell surface membrane, and 2. receptor-mediated endosomal entry [71]. The latter involves the formation of endosome and subsequent fusion of the viral and endosomal membrane, similar to the process described in ASFV. Thus, antivirals targeting endosomal entry, some of which have already been tested against SARS-CoV-2, could potentially be used for ASFV treatment. Both entry models begin with the interaction between the spike protein and the host receptor ACE2. Understanding viral entry and identifying the viral antigens and host receptors essential for infection present therapeutic opportunities for COVID-19 interventions. For example, monoclonal antibodies can prevent host attachment and small inhibitors can target host proteases involved in viral fusion to the host membrane [72]. In contrast, the viral factors necessary for ASFV attachment and entry have not been definitively determined. Therefore, identifying virulence factors should be prioritized in ASFV research, to advance the development of an effective ASF vaccine and antiviral therapeutics. Given the transmission between wild boars and domestic pigs, understanding immunology and viral entry into wild boars is crucial for developing a vaccine that can protect the wild boar population from ASFV infection.

Transmission routes play a pivotal role in disease control and management. The high transmission rate and the presence of asymptomatic infections in humans (approximately 40%) contributed to the rapid global spread of COVID-19, leading to a pandemic [73]. The primary route for COVID-19 is through respiratory droplets [15]. To mitigate the spread of the disease, numerous control measures such as social distancing, lockdowns, quarantine, and sanitization, have been implemented. Similar control measures have been employed for ASFV, including strict biosecurity measures, early detection and isolation of infected pigs, and sanitization of farms and cold-chain transportation. Culling infected or exposed animals is also necessary, to control the spread of ASFV, partly due to limited knowledge about the precise infectious dose and persistent viability of ASFV in various transmission routes. To minimize the amount of culling required, it is therefore crucial to determine these factors and take them into consideration. However, preventing and controlling the spread of ASFV may be more challenging than SARS-CoV-2, due to the virus’s ability to transmit through contaminated feed, pork products, and fomite. Additionally, ASFV has a relatively high estimated reproductive number (18) for a moderately virulent strain [74], compared with SARS-CoV-2 (2.87) [75], which further complicates disease control and management. These challenges in ASFV disease management emphasize the importance of effective diagnostic tools and systematic disease surveillance, which will be discussed later in this review.

To identify knowledge and technological gaps in the immune response against ASFV, we have summarized the similarities between immunomodulation studies conducted on SARS-CoV-2 and ASFV. By comparing these two viruses, we can identify potential biological targets, viral antigens triggering immune responses, technologies, and study designs that have been successfully employed in SARS-CoV-2 research. These findings and developments could potentially be applied to ASFV studies (see Table 1). The invasion of innate and adaptive immune responses in SARS-CoV-2 infection has been recently discussed [76]. While the primary sensing mechanisms differ between ASFV and SARS-CoV-2, there are shared downstream signaling factors and pathways. A summary of the modulation of these common signaling pathways in both viral infections is presented in Table 1. This information could be valuable for developing inhibitors targeting viral factors involved in these processes.

Understanding adaptive immunity against viral infection is paramount for disease control and vaccine design. Extensive investigations have been conducted on SARS-CoV-2, including the rapid identification of antigenic epitopes and the evaluation of immune responses and protection. In contrast, the immunological response to ASFV has primarily relied on experimental infections in domestic pigs and wild boars. While studies on SARS-CoV-2 utilized various methods such as in vitro assays, case-control studies, and cross-sectional and cohort studies to characterize T cell responses in humans [77], similar correlation studies between T-cell responses and symptoms in ASFV have not been established. CD4^+^ and CD8^+^ T cells play important roles in the response to viral infections. In SARS-CoV-2, CD4^+^ T cells differentiate into helper and effector cells that facilitate B cell maturation, release cytokines, assist CD8^+^ T cell, or aid in tissue repair [78]. Cohort studies on SARS-CoV-2 have shown a correlation between CD4^+^ cell response and milder symptoms, whereas the presence of CD8^+^ T cells has been associated with better prognosis, although less consistently [79,80,81]. In patients with milder symptoms, the activation of a sub-population of T helper cells and regulatory T cells has been observed, while aberrant adaptive responses have been linked to critically ill patients. Regarding ASFV, CD8^+^ cell response has been associated with partial protection in domestic pigs, but CD4^+^ responses remain understudied [53]. Identifying viral antigens as T cell epitopes in ASFV and understanding their impact on immune responses in susceptible hosts could contribute to the development of effective ASF vaccines. Despite the in-depth studies on T-cell responses in SARS-CoV-2, the durability and contribution of T-cells in preventing reinfection remains to be elucidated, similarly to the case of ASFV.

**Table 1:** Viral entry and immune modulation by SARS-CoV-2 and ASFV.

Features	SARS-CoV-2	ASFV
**Viral entry mechanism**		
**1. Cell surface entry**		
1.1 Required viral factors	Spike (S) [66]	Unknown
1.2 Required host factors	ACE2 [66], TMPRSS2 [82]	Unknown
**2. Receptor-mediated endocytosis**		
2.1 Required viral factors	S [67]	p12 [83], p30, p54 (E183L), and p72 [23,30,66,83,84], EP248R and E199L [85]
2.2 Required host factors	ACE2 [67], Cathepsin L [86]	CD163 [27,29], CD45 [28]
**3. Antibody dependent enhancement**		
3.1 Required viral factors	S [87]	Unknown
3.2 Required host factors	FcγR [87]	Fc-receptor [88]
**4. Macropinocytosis**		
4.1 Required viral factors	Unknown	Phosphatidylserine on viral surface [89]
4.2 Required host factors	Unknown	AXL [89]
		
**Immune modulations**		
**1. Major viral sensing pathway**	RIG-I-like [90] and Toll-like receptors [91] (RLRs and TLRs)	cGAS-STING [92]
**2. Common mechanisms in modulating innate immune response**		
2.1 Blocking activation of IRF3	Nsp1 [93]	pMGF505-15R [94] and pE120R [37], pMGF360-11L [95]
2.2 Promote degradation of IRF3	Nsp3 [96], Nsp5 [96,97]	pMGF360-14L [98], pM1249L [99]
2.3 Blocking nuclear translocation of IRF3	Nsp1 [93], Nsp5 [100], Nsp12 [101], ORF3b [102]	pMGF505-7R [94], pA137R [100,103]
2.4 Blocking nuclear transport by targeting importins	ORF6 [104], Membrane (M) [105]	pMGF360-12L [39]
**3. Common mechanisms in modulating adaptive immune responses**		
3.1 Viral antigens recognized by T lymphocytes	S, M, N, and NSPs [106]	pCP204L [107], pB646L [108], k11L [109], B646L, CP204L, I73R, MGF110-5L, CP530R, I73R, I215L, A151R, C129R, E146L, L8L, M448R, MGF110-4L [110], EP153R and EP402R [111], MGF100-1L, MGF505-7R and A238L [112], pMGF505-7R, pA238L, and pMGF100-1L [112]
3.2 CD8^+^ cell response	Yes [79,80,81,113]	Yes [114,115,116,117]
3.3 CD4^+^ cell responses	Yes [79,80,81]	Yes [116,117]
3.4 Secretion of cytokines	Yes [118,119,120,121]	Yes [122,123,124]
3.5 Activation of γδ T cell responses	Yes [125,126,127]	Yes [128,129,130]

## 3. Viral Detection

### 3.1. Current Status and Progress of ASF Research

Due to the severity and high mortality of ASF, as well as the lack of effective vaccines and antiviral drugs, rapid and precise detection of ASFV in pigs is crucial for disease control and prevention of economic losses.

According to the World Organization of Animal Health (WOAH), infection with ASFV is defined by the isolation of ASFV from a sample of suid, or the detection of ASFV-specific antigens, nucleic acids, or antibodies in samples from a suid showing clinical signs or pathological lesions consistent with ASF, or epidemiologically linked to a case of ASF [131]. Based on this definition, detection of ASFV can be based on the identification of live viruses, antigens, nucleic acids, or antibodies. Currently, real-time quantitative PCR (qPCR) is the preferred and most reliable method for ASFV detection. It offers high sensitivity, specificity, and rapid results, making it the gold standard for ASF diagnosis. However, qPCR requires specialized equipment and laboratory skills, thereby limiting its feasibility for on-farm use. On the other hand, most of the commercially available diagnostic kits for ASFV employ enzyme-linked immunosorbent assay (ELISA) to detect the presence of ASFV antigens or antibodies against the virus in samples. While more easily deployed on the farm, ELISA suffers from a relatively lower sensitivity and sometimes cannot distinguish between cases of active infection and past infection, when antibodies against the virus are still present. In addition, unlike nucleic acid-based diagnostics, antibody detection cannot differentiate between different ASFV genotypes.

Over the past few years, numerous detection methods for ASFV have been developed, including the detection of viral nucleic acids, antigens, as well as antibodies with improved sensitivity and ease of deployment (Table 2). Advances in instrumentation, such as a handheld fluorescence detector, portable PCR machine, and integration with microfluidic devices have also helped simplify ASF diagnosis. Notably, many of the new nucleic acid detection methods circumvent the equipment requirements of PCR by employing isothermal amplification methods, such as recombinase polymerase amplification (RPA) and loop-mediated isothermal amplification (LAMP), and in some cases, signal amplification using CRISPR-Cas systems. These highly sensitive and specific methods, if made available at an affordable price, could have great potential for ASF control and prevention of economic loss. We note, however, that in most of these studies, the assays could not be directly performed using clinical samples; a nucleic acid extraction step was still required. Since the studies utilized different clinical samples and sample preparation methods, their reported performances cannot be directly compared and are not necessarily an indication of how well they would perform in real-world field settings.

### 3.2. Lessons Learned from COVID-19 Research

Since the emergence of COVID-19, various diagnostic methods have been introduced, such as viral culture, chest imaging, antibody tests, rapid antigen tests, and CRISPR-based nucleic acid detection methods, such as LAMP-CRISPR and RPS-CRISPR. Among these technologies, the RT-PCR test stands out as the most reliable and is considered the gold standard. It identifies the virus’s genetic material from swabs taken from the nose or throat and is highly accurate. However, it demands specialized equipment and expertise, with results taking several hours to days. For faster results in remote areas or household settings, rapid antigen tests are commonly utilized. These tests quickly identify specific viral proteins from nasal swabs but might be less sensitive than RT-PCR. When the results are ambiguous or symptoms suggest an infection, chest imaging helps spot lung changes typical of COVID-19.

Compared with those for COVID-19, the diagnostic methods for ASF have been developed using similar strategies, including the detection of nucleic acid by PCR or isothermal amplification such as LAMP and RPA, with or without CRISPR-mediated signal modification, as well as detection using antigen–antibody interactions. In addition, diagnostic methods for both diseases have been continuously updated and improved, taking into account new insights in viral biology, structural biology, and genome sequencing of new viral strains, in order to identify new diagnostic targets, enhance detection sensitivity, and differentiate between various viral strains.

However, as described in the above section, there are also substantial differences in the biology and viral transmission between the ASFV and SARS-CoV-2, leading to various challenges in detecting ASFV that need to be overcome. One notable difference is the genome size, with ASFV having a genome more than six times the size of that of SARS-CoV-2 and containing a significantly higher number of genes. This diversity offers possibilities for new potential diagnostic targets. Moreover, ASFV circulates and reaches high viral loads in blood, whereas SARS-CoV-2 primarily accumulates in the respiratory tract. Thus, sample collection for ASFV detection currently involves blood sampling, which is more invasive compared with the nasopharyngeal swap performed in SARS-CoV-2 detection. Despite the development of different diagnostic methods, however, all currently commercially available ASF diagnostic kits are based on either PCR or the detection of specific viral antigens p30, p54, p72, and pp62 or antibodies against these proteins using ELISA. Furthermore, the ELISA detection kits exhibit limited sensitivity, even with the detection of p30, which is considered the most effective. Consequently, the test results from ELISA kits often require confirmation by PCR, to ensure accuracy [171].

For effective ASFV surveillance, a detection method must be capable of identifying both low-virulence and high-virulence strains. The low-virulence strains, like their high-virulence counterparts, can quickly spread through the pig population. These strains often cause slow growth, without obvious clinical symptoms, preventing timely detection and disease isolation [172]. The ability to detect these low-virulence strains would therefore be highly valuable for disease surveillance. In addition, the newly detected low-virulence strains also have the potential to be developed into live attenuated vaccines.

ASF causes infection in livestock, unlike COVID-19 that infects human population. Therefore, any detection technology adopted should be able to test thousands of samples at low cost. Rapid antigen tests, such as the lateral flow devices available for both ASFV and SARS-CoV-2 detection, offer low-cost and rapid point-of-care testing. However, their sensitivity is relatively low (approximately 100,000–1,000,000 copies/mL of viral genome are required [173,174]) compared with PCR. The success of lateral flow test application in SARS-CoV-2 detection and disease control is due to the short incubation time of the disease and high viral loads in the nasal cavity and saliva, which can be easily collected for rapid tests. On the other hand, collecting samples with a high ASFV viral load from infected or exposed pigs (i.e., blood or serum) for rapid ASFV testing remains a major burden for on-farm application. To overcome this, alternative point-of-care methods employing mobile real-time PCR or isothermal amplification, with or without signal amplification using CRISPR-Cas, offer greater sensitivity for detection. These methods may allow non-invasive sample collection, such as from saliva, and thereby would greatly simplify surveillance efforts. However, it is important to note that these alternatives come with higher costs, due to the need for specialized equipment for DNA amplification and extraction. To address this, researchers should explore non-invasive or less stressful methods of collecting blood samples with high ASFV viral loads from pigs. In addition to optimizing sample collection methods, it is crucial to develop appropriate sampling schemes for routine testing on farms. This would enable the timely detection of ASFV, allowing for more effective disease control measures.

## 4. Vaccines

### 4.1. Current Status and Progress of ASF Research

The global impact of ASFV infection on pigs is extensive, leading to widespread damage. Unfortunately, there are no existing vaccines or treatments. As a result, millions of infected pigs either die or need to be culled to prevent the spread of the disease, resulting in severe economic losses [175].

Historically, there have been many diseases in domestic pigs with high mortality rates. In many of these cases, vaccination has proven to be a very effective means of disease control. For example, classical swine fever, also known as hog cholera, has a mortality rate close to 100% in unvaccinated animals [176]. However, when pigs are vaccinated at least 10 days before challenge, the mortality rate drops to only 20%. Furthermore, the mortality rates in contact animals are even lower [177].

Therefore, considerable efforts have been made to develop vaccines against ASF [178]. Unfortunately, despite ongoing endeavors, there is currently no effective vaccine available for the disease. The current state of ASF vaccine development, including the approaches utilized, such as the use of inactivated or attenuated viruses, recombinant viral vector, and subunit vaccines, as well as the challenges faced, are further described below.

#### 4.1.1. Inactivated Viruses

Virus inactivation is an established method for vaccine production. The procedure is relatively simple, and vaccines produced through this method typically have excellent safety profiles. Inactivation prevents the vaccine from reverting to virulence and transmission, which can occur with some live attenuated viruses. However, despite numerous efforts, inactivated viruses have failed to elicit satisfactory protection against ASF, even with the use of advanced inactivation technologies, improved adjuvants, and high doses of inactivated viruses [179,180,181]. In many cases, the inactivated viruses were capable of inducing antibody responses in vaccinated animals. However, the observed response was not sufficient to confer disease protection. Furthermore, some vaccinated animals actually showed an accelerated clinical course, suggesting antibody-dependent enhancement of the disease [179]. Based on these observations, it has been suggested that using inactivated viruses might not be a suitable approach for ASF prevention and that further studies should focus on the neutralization ability and cellular immune response rather than solely relying on antibody titers when evaluating the potential of candidate vaccines.

#### 4.1.2. Live Attenuated Viruses

The use of live attenuated viruses circumvents many of the problems with inactivated viruses. Once introduced into pigs, live attenuated viruses mimic natural infection and can replicate within the host. This process stimulates both the humoral and cellular immunity pathways that are believed to be required for protection, without the need of adjuvants. There are three main groups of live attenuated viruses that can be used as vaccine candidates: naturally attenuated viruses, viruses attenuated by cell passages, and gene-deleted viruses. The European Commission has recently assessed the use of live attenuated strains as the most promising approach for developing effective ASF vaccines [182], as they have shown the most significant protection against challenge when compared with other ASF vaccines tested to date. Numerous efforts have been made to develop attenuated viruses as vaccines, and these have been extensively covered in various reviews [183,184,185,186,187]. In Table 3, we focus specifically on live attenuated viruses that have been tested against the live virus, to measure their protective capabilities in pigs.

Naturally attenuated viruses refer to ASFV strains found in nature that exhibit reduced virulence and/or reduction or loss of the hemadsorbing (HAD) phenotype. Examples of naturally attenuated ASFV strains that have been assessed as vaccines include NH/P68, OURT88/3, and LV17/WB/Rie1 (Table 3). Although these strains offer at least partial protection against future challenges with virulent strains, some of the vaccinated animals showed adverse reactions, including fever and skin lesions at the site of injection.

Other live attenuated viruses that have been tested as vaccines for ASFV include those that have been attenuated using cell passage and gene-deleted viruses. Several ASFV strains that have been passaged in either primary culture or continuous cell lines exhibited a reduction or loss of virulence and can sometimes confer protection against challenge by homologous virulent strains (Table 3, see [183] for a comprehensive review). These attenuated viruses have subsequently been characterized and found to have many gene deletions that could be related to the reduced virulence. These genes were then deleted from several ASFV strains, to produce gene-deleted viruses that were used as vaccine candidates (Table 3). Similarly to the naturally attenuated strains, however, viruses attenuated through cell passage and gene deletion often only confer protection to homologous virulent strains. In addition, the attenuated strains can sometimes revert to virulence and cause disease in immunized animals.

#### 4.1.3. Subunit, DNA, and Virus-Vectored Vaccines

Subunit, DNA, and virus-vectored vaccines offer potential solutions to many of the limitations associated with the use of live attenuated viruses, such as adverse side effects, reversion to virulence, and the lack of a stable cell line for live vaccine production. Several attempts have been made to use protein subunits [30,218,219,220,221], DNA [25,222,223], and viral vectors [221,224,225,226,227] as vaccine candidates, to induce immune protection against ASFV. In addition, the use of DNA-protein combinations [26,228], as well as heterologous prime-boost vaccination schemes [221,225,229], has also been explored. All the aforementioned studies, however, showed limited success and often had inconsistent results, likely due to the currently limited understanding of ASFV biology and virulence factors. Moreover, the variations in antigens, vaccination strategies, challenge methods, and methods for evaluating protection across different studies have hindered direct comparison of vaccine effectiveness. Nevertheless, these studies revealed that, in addition to inducing specific and neutralizing antibodies, T-cell response is also crucial for protection against ASFV challenge and should be evaluated in future vaccine studies [114,220,222,223,224]. Examples of subunit, DNA, and viral vector vaccines that have been investigated are provided in Table 4.

### 4.2. Lessons Learned from COVID-19 Research

During the COVID-19 pandemic, we witnessed vaccines being developed at an unprecedented speed. Since the first vaccines were made available in 2020, the rates of severe illness and mortality from COVID-19 have significantly decreased [232,233]. This achievement was made possible through international collaboration between governmental and private sectors, with the sharing of research infrastructure, expertise, and new research data.

The success of COVID-19 vaccine development relied on decades of progress in viral immunology, structural biology, protein engineering, and vaccine research, with special emphasis on previous coronaviruses such as SARS and MERS. Studies of viral surface proteins, including those of closely related viruses, and how they are recognized by protective antibodies, afforded a new understanding of how viral surface proteins should be maintained in a native conformation that can be targeted by neutralizing antibodies [14]. In addition, research on both traditional and novel vaccine platforms has enhanced our understanding of the factors affecting elicited immunity. Since different vaccine platforms offer distinct advantages, a strategic decision was made to invest in a variety of approaches during the COVID-19 pandemic. These included traditional methods such as whole inactivated virus and protein subunit vaccines, as well as newer vaccine platforms such as viral vector and nucleic acid vaccines. Notable examples of successfully developed COVID-19 vaccines currently in use include whole inactivated virus vaccines, e.g., CoronaVac by Sinovac and BBIBP-CorV by SinoPharm; protein subunit vaccines, e.g., Covovax™ by Novavax; non-replicating viral vector vaccines, e.g., ChAdOx1-S- (AZD1222) by AstraZeneca and University of Oxford, and Ad5-nCoV by CanSino Biological Inc. and Beijing Institute of Biotechnology; and mRNA vaccines, e.g., mRNA-1273 (Spikevax) by Moderna, and BNT162b2 (Comirnaty) by Pfizer/BioNTech.

Unlike SARS-CoV-2, ASFV is not only the sole virus in the genus *Asfivirus*, but also the only one in the entire Asfarviridae family. Due to its unique characteristics, scientists face huge challenges when attempting to apply or extrapolate information from studies into other viruses. Despite some studies on the structural biology and cell biology of ASFV, our understanding of viral recognition, entry, and immunology in pigs remains limited and subject to controversy. Furthermore, since ASFV primarily infects domestic pigs, wild boars, warthogs, and bush pigs, but not commonly used mammal model organisms such as mice or rats, knowledge about host–virus interactions, especially in vivo, is largely underdeveloped. This aspect adds another layer of complexity to our comprehension of the virus and how we should approach vaccine development and other related areas.

The fast and successful development of COVID-19 vaccines was largely facilitated by our accumulated knowledge of the virus and closely related ones. Therefore, it is reasonable to believe that, if we possessed sufficient knowledge about ASFV, we could apply similar expertise and platforms to develop vaccines against the virus. When we have gathered enough information, this would open the possibility of utilizing successful technologies such as mRNA vaccines, which have proven highly effective in preventing COVID-19 [234,235]. Since these promising technologies have not yet been employed in ASF vaccine development, this makes these areas worthy of further exploration.

## 5. Therapeutics and Drug Development

### 5.1. Current Status and Progress of ASF Research

The ASFV was discovered early in the 20th century, and its outbreaks have led to significant economic losses in the global swine industry. Vaccines have been considered the most effective preventive measure, while therapeutic drugs are crucial for reducing severe losses in case of infection. However, to date, most developed ASF vaccines have proven ineffective in inducing swine immune protection [236]. Consequently, there is a pressing need for the development of new antiviral drugs, and ongoing research in the field of ASFV aims to identify new drug targets and explore potential treatments [237]. This section will provide updates and discussions on recent antiviral agents against ASFV and newly identified drug target molecules, categorized according to their respective target molecules.

#### 5.1.1. Inhibitor Targeting Crucial Molecules Involved in Genetic Replication and Transcription

Several nucleoside or nucleotide analogs have shown potent antiviral activity [2]. For ASFV, iododeoxyuridine was the first nucleoside analogue identified in 1965 as inhibiting ASFV, but its use was limited due to a high cytotoxicity [238]. (S)-(3-hydroxy-2-phosphonylmethoxypropyl) (HPMP) nucleoside derivatives, such as (S)-1-(3-hydroxy-2-phosphonylmethoxypropyl)cytosine (HPMPC) or cidofovir, have demonstrated potent activity against various viruses [238]. However, nephrotoxicity was observed in vivo during a phase I/II clinical trial with cytomegalovirus (CMV)-infected patients, making the drug unsafe as an antiviral agent. Nonetheless, a cyclic derivative of HPMPC called HPMPC prodrug has been synthesized, which retains the same potency as HPMPC but with lower toxicity in rats [239]. The antiviral activity of the cyclic HPMPC derivative (cHPMPC) against ASFV has been demonstrated in recent in vitro and in vivo studies [240]. It showed dose- and strain-dependent activity against ASFV, with an IC_50_ below 1 µM in vitro and low cytotoxicity (CC_50_) against porcine bone marrow-derived macrophages at a concentration of 40 µM. Oral administration of cHPMPC to ASFV-infected pigs (Georgia 2007/1 strain) at a dose of 30 mg/kg body weight resulted in significantly reduced viral load in blood and tissues (spleen and lung) and delayed clinical signs compared with the control group. The target of cHPMPC is speculated to be viral DNA polymerase or the RNA polymerase involved in late transcription. While cHPMPC shows promise as an anti-ASFV agent, its efficacy may vary slightly among different ASFV strains. Further in vivo studies with other ASFV strains could provide valuable insights.

The identification of five putative RNA helicases (Q706L, QP509L, A859L, D1133L, and B962L) in the ASFV genome has drawn attention, as potential targets for helicase inhibitor screening [241]. Resveratrol and oxyresveratrol, which are stilbene compounds, have been found to inhibit ASFV replication in vitro, possibly by targeting a helicase. These compounds have shown effectiveness both as synthetic compounds and as extracts from mulberry twigs. At concentrations of 10 µg/mL for resveratrol and 30 µg/mL for oxyresveratrol, viral DNA synthesis was reduced by approximately 10- and 7.1-fold, respectively [241]. The effective inhibition observed with mulberry extract suggests the possibility of using natural extracts in feed mixes, providing a cost-effective approach to ASFV treatment and control. A specific drug screening targeting the ASFV D1133L helicase, crucial for virus replication, was later reported [242,243]. Among the screening compounds that bind to the D1133L helicase, periactin was found to inhibit ASFV replication, with minimal toxicity to porcine alveolar macrophages (even at 50 μM). Periactin was shown to inhibit the transcription and expression of ASFV structural proteins p30 and p72, which are required for the replication process of ASFV. In contrast, resveratrol only affected the expression of p72 [241]. In addition, periactin exerted an inhibitory effect on D1133L expression [244]. Another potential target is ASFV topoisomerase II, an enzyme essential for ASFV replication, as confirmed by a siRNA experiment [245]. Fluoroquinolones, bacterial topoisomerase inhibitors, were also found to be effective inhibitors against ASFV topoisomerase II [246]. Another ASFV topoisomerase II inhibitor, genistein flavonoid, disrupted viral DNA replication and resulted in viral DNA fragmentation [247]. As ASFV can disrupt host epigenetics by altering histone acetylation/deacetylation, histone deacetylase inhibiting compounds, such as sodium phenylbutyrate, function as ASFV inhibitors. This compound abolished viral replication and showed synergistic antiviral effect with ASFV topoisomerase inhibitor (enrofloxacin) [248]. Recently, compounds targeted at enzymes involved in nucleotide biosynthesis were found to exert anti-ASFV activity. The treatment of brequina, a dihydroorotate dehydrogenase inhibitor, for ASFV-infected Vero cells could inhibit viral infection, with an IC_50_ of 2.83 µM [249]. Nucleoside/nucleotide analogs remain attractive for ASFV inhibition and have shown the most progress in terms of in vivo supporting data. However, their high cost limits their practical use by farmers.

#### 5.1.2. Inhibitors Targeting Endosomal and Viral Entry, and Transport Pathways

Targeting the endosomal and viral entry pathways has shown promise for developing versatile antiviral drugs, as endocytosis is a common entry pathway essential for infection of pathogenic viruses. FDA-approved compounds such as Tetrandrine (TETR), Verapamil (VER), Apilimod (APL), Raloxifene (RLX), and Tamoxifen (TMX) have demonstrated efficient inhibition of ASFV, SARS-CoV-2, and Ebola pseudoviruses, as they share a common endocytic pathway. These compounds have exhibited IC_50_ values below 4.5 μM, with APL and TETR being the most potent inhibitors. They achieved over 99% reduction in pseudotyped SARS-CoV-2 entry and 80–90% reduction in ASFV infectivity. The mechanism of action for RLX and TMX involves altering free cholesterol accumulation and calcium flux within host cells [241]. Additionally, host cell microtubules play a role in ASFV entry, transportation, and replication. The antiviral compound 6b, a microtubule stabilizing agent, has shown effectiveness in inhibiting ASFV replication. The compound exhibited an IC_50_ value of 19.5 μM, indicating its potency in inhibiting ASFV replication. It demonstrated a low toxicity to cells, with a CC_50_ value greater than 500 μM, indicating minimal harm to cellular function. Furthermore, it showed low toxicity in animals, with a safe dosage up to 100 mg/kg. It acts in multiple stages, interfering with virus entry, inhibiting viral factory formation, and impeding progeny virus release [250]. An apigenin flavonoid derivative, named genkwanin, was found to inhibit a highly virulent ASFV strain (BA71V) in vitro (IC_50_ at 2.5 µM). It was speculated to bind to the tubulin molecule at the colchicine-binding site, resulting in the disruption of the tubulin polymerization essential for the viral egress pathway [251]. It is possible that, not only do drugs directly target viral molecules, but drugs impairing host factors required by the virus life cycle are also effective in inhibiting ASFV. It is worth noting that, while these compounds have demonstrated antiviral activity in vitro, their efficacy against ASFV in vivo has not yet been established.

#### 5.1.3. Inhibitor Targeting Proteases

Proteolytic processing of viral proteins is crucial for the replication of both DNA and RNA viruses [252]. Therefore, there is considerable interest in developing antiviral agents that target viral proteases. In the case of ASFV, the pS273R protease has been identified as a SUMO-1 specific cysteine protease that plays a role in the cleavage of polyprotein precursors, namely P220 and P62. These cleavages are essential for the maturation and infectivity of ASFV [12,253,254]. Inhibiting the activity of pS273R not only prevents the cleavage of P220 and P62, thus inhibiting ASFV replication, but also leads to an increase in cell immune factors [255,256]. Structure-based drug screening has identified the cysteine protease inhibitor (E-64) as a potent pS273R inhibitor, in which the E-64 forms a covalent adduct with the cysteine in the pS273R catalytic triad. In vitro tests with porcine alveolar macrophage (PAM) cells showed that E-64 had low cytotoxicity at a concentration of 4 mM. Furthermore, structure-based virtual screening assessed using the low Gibb binding free energy of pS273R with FAD-approved drugs has identified potential compounds that specifically bind to the protease, including leucovorin, carboprost, protirelin, flavin mononucleotide, and lovastatin acid. However, these compounds have not yet been evaluated for their anti-ASFV activity in vitro or in vivo [257].

#### 5.1.4. Inhibitors Targeting Other Proteins and Pathways

Recently, chlorine dioxide (ClO_2_) has emerged as a highly effective biocide for inhibiting ASFV in a PAMs infection model. ClO_2_ is a strong oxidant known for its antibacterial, antifungal, and antiviral properties and is commonly used for disinfection purposes in various settings, such as wastewater treatment, the food industry, and environmental sanitation [258,259]. Compared with other chemical oxidants, ClO_2_ exhibits relatively low toxicity [260]. ClO_2_ demonstrated significant inhibition of ASFV infection, with an IC_50_ of 0.08 µg/mL, while cytotoxicity was observed at a higher concentration (CC_50_ = 0.56 µg/mL). The antiviral mechanism of ClO2 involves blocking viral attachment and causing damage to viral nucleic acids and proteins. This mode of action is consistent with its inhibitory effect observed against porcine reproductive and respiratory syndrome viruses [259]. Given its effective anti-ASFV activity and cost-effectiveness, ClO_2_ holds promise for the treatment and prevention of ASFV in pig farms. Additionally, kaempferol, a potent flavonoid compound, has been identified through cell-based screening among a collection of more than ninety flavonoids [261]. Kaempferol exerts its antiviral effects by inducing autophagy in ASFV-infected Vero cells, thereby disrupting the ASFV replication cycle and inhibiting viral protein and DNA synthesis.

#### 5.1.5. Potential Drugs Targeting ASFV Protein–Protein Interactions

A recent study reported the discovery of potential drugs for ASFV by targeting swine-ASFV protein–protein interactions [262]. Through computational prediction, the study identified a protein–protein interaction network involving key proteins such as heat-shock proteins 90s (HSP90AB1, HSP90AA1, and HSP90B1) and TNF, which interact with various ASFV and swine proteins. Several known drugs, including Geldanamycin (DB02424), Polaprezinc (DB09221), and Andrographolide (DB05767), were suggested for targeting HSP90AA1, HSP90AB1, and HSP90B1, potentially disrupting the swine-ASFV protein–protein interaction network and inhibiting viral infections However, further confirmation is needed to establish the anti-ASFV activity of these compounds targeting protein–protein interactions.

### 5.2. Lessons Learned from COVID-19 Research

Efforts to find drugs against ASFV have yielded several effective compounds with demonstrated antiviral activity in vitro. Only a few of these potential antiviral compounds, such as the cHPMPC nucleoside analog, however, have shown efficacy against ASFV in vivo. Furthermore, significant obstacles hinder the practical use of ASFV drugs, including the challenge of ensuring efficacy against various viral strains, affordability for farmers, and consumer safety regarding drug residues in pork products. To expedite the discovery of effective and safe therapeutic drugs for emerging diseases, repurposing FDA-approved or existing drugs with an established safety profile in clinical trials is a widely embraced approach [263]. Repurposing FDA-approved drugs has proven successful in addressing emerging diseases, as seen with the use of favipiravir and dexamethasone for COVID-19 [264]. Favipiravir is a nucleoside/nucleotide analog approved for influenza treatment. It effectively inhibits viral RNA synthesis and demonstrated potency against yellow fever, enterovirus, Ebola, norovirus, and chikungunya prior to its use as a drug for COVID-19 [264]. Dexamethasone and other corticosteroids are commonly prescribed to reduce inflammation and suppress the immune system. These drugs are used to treat a range of conditions, including allergic reactions, asthma, rheumatoid arthritis, autoimmune disorders, and certain types of cancer. When administered systemically, they have been shown to improve the survival rate of hospitalized COVID-19 patients requiring oxygen therapy [264]. Furthermore, extensive efforts are underway to identify repurposed drugs that target new biomolecules, such as the M^pro^ protease of SARS-CoV-2 [252,265]. This approach provides a promising avenue for discovering therapeutic drugs for COVID-19 treatment. In conclusion, numerous FDA-approved drugs are currently undergoing active research and being explored as potential treatments for COVID-19. It is hoped that, similarly to COVID-19, repurposed drugs can also be employed to control outbreaks of ASF. However, there is an additional concern when it comes to ASFV drugs: for a safe and effective drug to be widely implemented, it must also be priced at a level acceptable to farmers. If the cost is too high to treat all infected pigs, it should at least be affordable for farmers to treat their valuable and expensive breeders if they get infected. Nonetheless, the discovery of active compounds in herbs and medicinal plants may alleviate this concern by utilizing economically viable plant extracts in animal feed. We are making progress towards finding a well-balanced drug that effectively combats ASFV, ensuring both safety and affordability for farmers.

## 6. Disease Surveillance

### 6.1. Current Status and Progress of ASF Research

For a more effective and efficient healthcare system and better prevention and control of disease outbreaks, a surveillance system is required. This involves the continuous systematic collection of information about diseases in certain populations, as well as analysis and interpretation of data that are required for planning and implementing disease control activities. When performing well, small investments in surveillance can be effective in reducing disease, death, and economic losses, because they can lead to early detection of epidemics, thereby resulting in early control and prevention of diseases. Generally, surveillance can be classified into two categories: epidemiological surveillance and genomic surveillance [266,267]. Epidemiological surveillance includes rumor surveillance, monitoring of media sources, and informal and formal reporting networks inside and outside health facilities [268,269]. The current spread of ASF, while not comparable in scale of that of COVID-19, has been detected in several countries across Africa, Asia, and Europe. Because the epidemiological surveillance of ASF has been described in detail elsewhere [270,271,272,273,274], this review will focus only on the genomic surveillance of the disease, which has in some ways seemingly been neglected.

Genomic surveillance is the process of monitoring genetic sequences of pathogens, in order to understand their transmission, evolution, and potential impact on public health. It helps identify new variants and track their spread, providing critical information for controlling an outbreak. The process has become an important tool in the fight against infectious diseases and is used by researchers and public health officials to detect outbreaks, monitor the effectiveness of vaccines and treatments, and inform public health policies. Like all viruses, ASFV mutates as it replicates and spreads in a population, resulting in new viral variants that may have differences in phenotypes which may affect host–pathogen interactions [267,270,271,275]. Several strains of ASFV have been sequenced and reported. However, systematic analyses and monitoring are unfortunately inadequate for controlling the disease, both locally and globally.

Since its initial identification in Kenya in the 1920s, ASFV has been reported in various regions worldwide. Strains of the virus have emerged in different areas, including Europe in 1957, China in 2018, and nearly all Southeast Asian countries since 2018 [276,277,278]. More recently, in 2022, ASFV was finally detected in Thailand. Despite the existence of multiple strains, many molecular characterization studies have only partially sequenced specific viral genes, such as p72. Based on the sequence homology at the carboxy terminus of the p72 capsid protein encoding gene, ASFV is classified into 24 genotypes [279,280]. Of all the 24 genotypes, so far, only genotypes I and II have been identified outside Africa [281,282]. Currently, only 229 complete genomes of ASFV have been sequenced and deposited in the NCBI nucleotide database (as of 16 July 2023). Unfortunately, only a few systematic efforts have been made to analyze the entire genome of local viral strains, and, to our knowledge, no comprehensive global systematic analysis has been conducted to date. This limited understanding of the virus evolution and epidemiology therefore poses a significant drawback for preventing and controlling ASF outbreaks.

### 6.2. Lessons Learned from COVID-19 Research

Since the COVID-19 outbreak emerged in December 2019, tremendous efforts have been made to rapidly understand more about the deadly disease through analyses of genomes of the causative virus SARS-CoV-2. As the virus spread rapidly, and due to the nature of its RNA genome, which is able to mutate at a fast rate, a number of variants have been identified. To date, over ten million strains of the virus have been sequenced and documented in public repositories such as Global Initiative on Sharing All Influenza Data (GISAID) [283,284]. These variants can be typically classified into four different types, based on how they may affect vaccines, therapeutics, and diagnostics, as well as the transmission and severity of disease: variant of interest (VOI), variant of concern (VOC), variant of high consequence (VOHC), and variants being monitored (VBM) [285,286]. First, VOI refers to a specific strain of the virus that has genetic changes or mutations compared with the original or predominant strain, and there is preliminary evidence to suggest potential implications for transmission, diagnostics, therapeutics, or vaccine effectiveness. VOIs are monitored closely, as they have the potential to become more prevalent or exhibit specific features that require further investigation. Second, VOC is a specific strain of the virus that has been identified as having increased transmissibility, altered disease severity, significant impact on diagnostics, reduced effectiveness of treatments or vaccines, or other concerning features. VOCs are closely monitored and often prompt a heightened public health response and additional measures to control their spread. Third, VOHC refers to a specific strain of the virus that is associated with severe disease outcomes, significantly increased hospitalizations or deaths, substantial immune escape, or a significant reduction in the effectiveness of public health measures or medical countermeasures. They are monitored closely because of their potential to cause severe public health impacts and may require specific interventions or response strategies. Last, VBM are strains of the virus that are under surveillance due to specific genetic changes or mutations, but they have not yet reached the threshold to be classified as a VOI or VOC. These variants are actively monitored through ongoing genetic surveillance efforts, to assess their impact on transmission, severity, diagnostics, treatments, and vaccines.

From the start of the COVID-19 outbreak, numerous international initiatives have been dedicated to collecting the genomic data of newly sequenced viral strains. These efforts involve organizations such as the World Health Organization (WHO), the United States Centers for Disease Control and Prevention (CDC), and the European Centre for Disease Prevention and Control. In June 2020, the WHO established the Virus Evolution Working Group to focus on SARS-CoV-2 variants, their characteristics, and impacts on countermeasures. In late 2020, the WHO began characterizing VOIs and VOCs, to prioritize global monitoring, research, and response adjustments. In May 2021, the WHO introduced simplified labels for key variants, to facilitate communication and disease control to the public. In March 2023, the WHO updated and published its current tracking system and working definitions for VOCs, VOIs, and variants under monitoring (VUMs). Many other global efforts are also underway to improve genome surveillance around the world, including AFRO-Africa Centre for disease Control, the Pan American Health Organization COVIGEN Network, Regional Genomic Surveillance Consortium from WHO Southeast Asia Region, and the ACT-A WHO Global Risk Monitoring Framework [287,288]. Moreover, there are also several web-based platforms providing up-to-date visualization of genomic data of SARS-CoV-2 and geographic distribution of variants such as Global Initiative on Sharing All Influenza Data (GISAID), Nextstrain, and outbreak.info [284,289,290,291]. This global system enables the public and authorities to swiftly assess the risk of new variants to public health and involves monitoring the viral spread in animals and chronically infected individuals, as there is concern about potential mutations and the emergence of harmful variants.

Enhancing global pandemic preparedness requires prioritizing the advancement of pathogen genomic surveillance efforts. However, numerous challenges have been observed during the genomic surveillance of SARS-CoV-2, hindering progress in this area. Although it is recommended that sequencing 0.5% of total confirmed cases within a turnaround time of less than 3 weeks serves as a benchmark for genomic surveillance studies targeting SARS-CoV-2 [288], many countries unfortunately have not been able to comply with this recommendation, for many reasons. Notably, only 38.1% of countries have conducted high-level routine genomic surveillance, while 14.4% have implemented a moderate level, 21.2% a low level, and 26.4% have a limited level of genomic surveillance [292]. Disturbingly, there is a lack of available data for some countries. Genomic surveillance strategies also vary globally, with many countries having limited surveillance capabilities. Furthermore, the diversity of genomic data properties and the low proportion of cases sequenced in most countries exacerbate the situation [288]. Additionally, many countries face legal limitations that prevent them from sharing genomic data in public repositories. Notably, low- or low-middle-income countries, as classified by the World Bank, are particularly lacking in genomic surveillance data, likely due to constraints in infrastructure capacity and resources [288,292]. Nevertheless, the ongoing genomic surveillance of SARS-CoV-2 is still an important tool in the fight against COVID-19 and is likely to continue to play an important role in SARS-CoV-2 research and treatment in the future.

Far greater than any other pathogens, genomic surveillance efforts for SARS-CoV-2 have been made at an unprecedented rate and are crucial for controlling the spread of COVID-19 worldwide. This information is valuable and indispensable for managing the current outbreak, as well as future ones. The technological advancements achieved so far can be applied, not only to human pathogens, but also to infectious diseases of animals such as ASF. Lessons can be learned from the challenges encountered during the COVID-19 crisis, such as the variations in the quality and quantity of genome sequencing among different research institutions and countries, as well as legal restrictions that hinder the sharing of sequenced data in certain nations. Therefore, to fully harness the potential of technology and the genomic surveillance approach in combating ASFV and other future infectious outbreaks, international collaboration and discussions between the public and governments are required. Such efforts will greatly enhance our ability to effectively combat future pandemics.

## 7. Conclusions and Perspectives

COVID-19 has had a profound impact as a devastating global viral pandemic, causing millions of deaths worldwide. However, amidst the tragedy, there is an unseen benefit in the form of valuable lessons that humanity can glean from this ongoing crisis, to better control and mitigate such devastating infections. Scientists from around the world are collaborating tirelessly to conduct research and development efforts aimed at combating disease. These endeavors encompass various aspects such as diagnostics, vaccine development, drug repurposing, and disease surveillance systems. Importantly, the knowledge and platforms established through these endeavors can be harnessed and applied to address not only the present but also future infectious diseases, encompassing both human and animal health.

Using the technological advancements gained from COVID-19 research in the context of ASF has the potential to strengthen protection and prevention efforts against this economically devastating porcine disease. However, it is important to acknowledge that not all aspects and technologies can be seamlessly transferred, due to the inherent differences between pathogens and their respective hosts. Therefore, extensive discussions among experts and all stakeholders are crucial, in order to navigate these challenges. International collaboration, involving both the private sector and governments, is also essential for effective implementation. By applying technological spillovers and the valuable lessons learned from COVID-19 to ASF and future emerging diseases, we can ensure that the huge losses incurred during the COVID-19 pandemic were not in vain.

## Figures and Tables

**Table 2:** Diagnostic approaches for the detection of ASFV.

Techniques	Target Genes/Protein	Samples and Preparation	Detection Signal	Sensitivity	Remarks
**Nucleic acid-based detection**					
**PCR**					
	B646L	B646L DNA spiked on swine blood/tissues	Lateral flow strip	15 copies/reaction	[132]
	B646L	Nucleic acid purified from blood	Fluorescence	25.6 copies/μL	Quadruple PCR-based for simultaneous detection of ASFV, CSFV, APPV [133]
	B646L/MGF-360I4L/CD2v	Nucleic acid purified from blood/fecal/tissue/floor swab	Fluorescence	47–82 copies/μL	[134]
	B646L	Nucleic acid purified by multiprobe assisted DNA capture from blood/tissue	Fluorescence	0.5 copies/μL	[135]
	B646L	Nucleic acid purified from brain/liver/lung/spleen	Fluorescence	643 copies/μL	Multiplex PCR for simultaneous detection of ASFV, CSFV, APPV [136]
**LAMP**					
	Conserved ASFV sequence	Nucleic acid purified from blood/tissue	Fluorescence probe with microfluidic chip platform	10 copies/μL	[137]
	B646L	B646L DNA spiked on swine tissue	Lateral flow strip	4 copies/reaction	[138]
	B646L	Nucleic acid purified from whole blood using filter paper dipstick	Fluorescence	10 copies/reaction	[139]
	Topoisomerase II	Serum, rectal/oral swabs without purification	Colorimetry	400 copies/reaction	(Hand-held portable amplification machine) [140]
	B646L	Nucleic acid purified from blood	Colorimetry (Naked eye)	5 copies/reaction	Improve sensitivity by self- replication catalyzed hairpin assembly [141]
	CD2v/MGF505-2R/p72	Nucleic acid purified from serum/tissues	Fluorescence/microfluidic-LAMP chip detection system	101 copies/μL	[142]
**RPA**					
	B646L	Nucleic acid purified from blood/serum	Lateral flow	50 copies/reaction	[143]
	B646L	Nucleic acid purified from blood/serum	Lateral flow	100 copies/μL	[144]
	B604L	Serum/blood with heat/lysis buffer extraction without purification	Fluorescence	3.5 copies/μL	[145]
**CRISPR**					
PCR-CRISPR					
	B646L	Serum, whole blood, processed tissue samples; direct PCR without nucleic acid extraction	Fluorescence biosensor; lateral flow strip	4 copies/μL for fluorescence biosensor; 40 copies/μL for lateral flow strip	[146]
	B646L	Plasmid, DNA extracted from pig whole blood	Lateral flow strip	2.5 fM	[147]
					
LAMP-CRISPR					
	B646L	Nucleic acid purified from whole blood using filter paper dipstick	Fluorescence	1 copy/reaction	[139]
	B646L	Plasmid, DNA extracted from pig blood	Fluorescence	7 copies/reaction	[148], single-tube reaction; LAMP reaction in the tube, CRISPR reagents in the lid to be subsequently mixed
	B646L	DNA extracted from pig nasal swab, spleen, liver, lung, submandibular lymph node and kidney	Fluorescence	7 copies/μL	[149], single-tube reaction; LAMP reaction in the tube, CRISPR reagents in the lid to be subsequently mixed
	topoisomerase	Whole blood spiked with plasmid	Fluorescence	N/A	[150], single-tube, CRISPR reaction was dehydrated on the lid
					
					
RPA-CRISPR and RRA-CRISPR					
	B646L	DNA extracted or prepared by room temperature lysis without extraction from pig serum	Fluorescence; lateral flow strip	20 copies/reaction for lateral flow strip	[151]
	B646L	DNA extracted from clinical samples	Colorimetric	100 copies/μL	[152]
	B646L	DNA extracted from pig nasopharyngeal swabs, blood, spleen, liver	Lateral flow strip	10 copies/reaction	[153]
	B646L	DNA from clinical samples	Fluorescence; lateral flow strip	3 copies/μL for fluorescence	[154], one-tube reaction
	DNA Pol; pp220	DNA extracted from pig blood, oral swab, anal swap	Fluorescence; lateral flow strip	200 copies/sample for lateral flow strip	[155]
	B646L	Pig serum treated with heat and denaturant	Colorimetric	N/A	[156]
	B646L; pig ACTB (internal control)	DNA extracted from whole blood	Fluorescence (smartphone-based hand-held device)	N/A	[157], multiplex detection in single tube using LbCas12a and LbuCas13a
	B646L	DNA extract from blood	Fluorescence	8 copies/reaction	[158]
	B646L	Pig serum after heat treatment and chemical reduction	Colorimetric	200 copies/reaction	[159], colorimetric reaction based on gold nanoparticle that can be observed with naked eye
					
					
**Protein-based detection**					
**Antigen**					
	p30 protein	Pig nose and mouth discharge	Time-resolved fluorescence immunoassay/double-antibody sandwich immunoassay	0.015 ng/mL	[160]
	p30 protein	Pig blood/tissues	Lateral flow immunoassay	N/A	[161]
**Antibody**					
	p30 antibody	Pig serum	Chemiluminescence	N/A	[162]
	p22 and p30 antibodies	Pig serum	Indirect ELISA	1:600 serum dilution	[163]
	p30 antibody	Pig serum	Nanoplasmonic biosensor	1:16,000 serum dilution	[164]
	pB602L antibody	Pig serum	Indirect ELISA	1:6400 serum dilution	[165]
	p30 antibody	Pig serum	Bioluminescence base immunoprecipitation	1:100 serum dilution	(Gaussia luciferase linked with p30 protein) [166]
	p54 antibody		Competitive ELISA (HRP conjugate nanobody)	N/A	[167]
	p72 antibody	Pig serum	Lateral flow immunoassay	1:10,000 serum dilution	[168]
	p17 antibody	Pig serum	Indirect ELISA	1:1280 serum dilution	[169]
	K205R antibody	Pig serum	Colorimetric competitive ELISA	1:128 serum dilution	[170]

N/A = Not applicable.

**Table 3:** Live attenuated ASFV strains that have been evaluated as candidate vaccines, and the associated genetic deletions.

Strain	Deleted/Mutated Genes	Challenge Strain	Protection (% Survival, N *)	References
**Naturally attenuated**				
NH/P68		Heterologous (L60)	0, 19	[188]
OURT88/3		Heterologous (Benin 97/1)	60–100, 9 ^#^	[189]
		Heterologous (Uganda 1965)	100, <12	[189]
		Homologous (OURT88/1)	50, 8	[190]
		Heterologous (DRC 085/10)	100, 4	[190]
		Homologous (OURT88/1)	67, 6 (intranasal); 50, 6 (intramuscular)	[122]
Lv17/WB/Rie1	CD2v (EP402R)	Homologous (Lv17/WB/Zieme3)	100, 2	[191]
		(Arm07)	92, 12	[192]
**Attenuated though cell passage**				
ASFV-G-ΔI177L/ΔLVR (Plum island porcine epithelial cells)	MGF360-6L, MGF300-1L, MGF300-2R, MGF300-4L, MGF360-8L, MGF360-9L, MGF360-10L N-terminus portion of MGF360-4L, C-terminus portion of MGF360-11L, X69R	Homologous (ASFV-G)	100, 5	[193]
Spencer passage 39 and 44 (primary pig kidney cell)	No sequencing data	Homologous (Virulent Spencer)	100, 4	[194]
Portuguese passage 34 (primary pig kidney cell)	No sequencing data	Homologous (Virulent Portuguese)	100, 2	[194]
Gasson passage 23 (primary pig kidney cell)	No sequencing data	Homologous (Virulent Gasson)	0, 2	[194]
Congo KK-262 (Attenuated Congo K-49 passaged in porcine kidney cell lines and porcine bone marrow cells),	No sequencing data	Homologous (Congo K-49)	80–100, 5–6 ^#^	[195]
France F-32/135 (attenuated France F-32 passaged in porcine bone marrow cells)	No sequencing data	Homologous (Congo K-49)	0–20, 4–5 ^#^	[195]
Hinde WH II (porcine buffy coat culture)	No sequencing data	Homologous (Hinde WH II)	45, 102	[196]
Ugandan (porince buffy coat culture)	No sequencing data	Homologous (Ugandan)	90, 5	[197]
ASFV-G passage 110 (Vero cell)	No sequencing data	Homologous (ASFV-G)	0, 5	[198]
Stavropol passage 33 (A_4_C_2_/9k cell)	No sequencing data	Homologous (Stavropol)	0, 2	[199]
Stavropol passage 20 (CV-1 cell)	No sequencing data	Homologous (Stavropol)	0, 2	[199]
E75 passage 4 (CV-1 cell)	No sequencing data	Homologous (E75)	100, 4	[200]
		Heterologous (BA71)	0, 4	[200]
**Gene-deleted**				
BA71ΔCD2v	CD2v (EP402R)	Homologous (BA71)	100, 6	[201]
		Heterologous (E75)	100, 6	[201]
		Heterologous (Georgia 2007/1)	100, 6	[201]
		Heterologous (RSA/11/2017)	83.3, 6	[202]
		Heterologous (Ken06.Bus)	33, 6	[202]
HLJ/18-7GD	MGF505-1R, MGF360-12L, MGF360-13L, MGF360-14L, MGF505-2R, MGF505-3R, and CD2v	Homologous (HLJ/18)	100, 4	[203]
ASFV-G-ΔI177L	I177L	Homologous (Georgia 2010)	100, 20 100, 10	[204] [205]
ASFV-G-ΔA137R	A137R	Homologous (Georgia 2010)	100, 5	[206]
ASFV-G-ΔE184L	E184L	Homologous (Georgia 2010)	100, 3	[207]
SY18ΔI226R	I226R	Homologous (SY18)	100, 10	[208]
OURT88/3ΔDP2	DP71L and DP96R	Homologous OURT88/1	66, 6	[209]
ASFV-G-Δ9GL	9GL (B119L)	Homologous (Georgia 2007)	100, 10	[210]
ASFV-G-Δ9GL/ΔUK	9GL (B119L) and UK (DP96R)	Homologous (Georgia 2007)	100, 10	[211]
ASFV-G-Δ9GL/ΔNL/ΔUK	9GL (B119L), NL (DP71L), and UK (DP96R)	Homologous (Georgia 2007)	0, 5	[212]
ASFV-G-ΔMGF	MGF505-1R, MGF505-2R, MGF505-3R, MGF360-12L, MGF360-13L, and MGF360-14L	Homologous (Georgia 2007)	100, 20	[213]
ASFV-G-Δ9GL/ΔMGF	9GL (B119L), MGF505-1R, MGF505-2R, MGF505-3R, MGF360-12L, MGF360-13L, and MGF360-14L	Homologous (Georgia 2007)	0, 5	[214]
BeninΔDP148R	DP148R	Homologous (Benin 97/1)	100, 15	[215]
BeninΔMGF	MGF360-9L, MGF360-10L, MGF360-11L, MGF360-12L, MGF360-13L, MGF360-14L, MGF530/505-1R, MGF530/505-2R, MGF530/505-3R, and MGF530/505-4R	Homologous (Benin 97/1)	100, 5 50–83, 6 ^#^	[40] [123]
ArmΔCD2v-ΔA238L	CD2v (EP402R) and A238L	Korean Paju	100, 4	[216]
ASFV-ΔQP509L/QP383R	QP509L and QP383R	CN/GS/2018	0, 6	[217]

* N denotes the number of pigs vaccinated and then exposed to live ASFV. ^#^ The % survival rate is presented as a range, since different doses of vaccine or live virus challenge were administered during the experiment.

**Table 4:** Subunit, DNA, and virus-vectored ASF vaccines.

Antigen/Gene ^1^	Antigen Strain	Adjuvant	Specific Antibodies	Neutralizing Antibodies	T-Cell Response	Challenge Strain	Protection (% Survival, N *)	References
**Subunit vaccines**								
CD2v (EP402R)	E75CV	Freund’s	Yes	partial	N/A	E75	100, 3	[218]
p30 (CP204L)	E75	Freund’s	Yes	Yes	N/A	E75	0, 3	[30]
p54 (E183L)	E75	Freund’s	Yes	Yes	N/A	E75	0, 3	[30]
p54+p30	E75	Freund’s	Yes	Yes	N/A	E75	50, 6	[30]
p54/p30 chimera	E75	Freund’s	Yes	Yes	N/A	E75	100, 2	[219]
p54+p30+p72+p22	Pr4	Freund’s	Yes	Yes	N/A	Pr4	0, 6	[220]
p72, p54, p12	Georgia 2007/1	TS6	Yes	N/A	partial	N/A	N/A	[221]
**DNA vaccines**								
p54/p30 fusion	E75		No	N/A	No	E75	0, 4	[25,222]
sHA ^2^/p54/p30 fusion	E75		Yes	No	Yes	E75	0, 6	[222]
Ubiquitin/sHA/p54/p30 fusion	E75		No	No	Yes	E75	25, 12	[222]
SLA-II/p54/p30 fusion	E75		Yes	No	Yes	E75	0, 4	[25]
Expression library containing ~80 ORF fused with ubiquitin	E75		Yes (after challenge)	N/A	Yes (after challenge)	E75	60, 10	[223]
**Virus-vectored vaccines**								
BacMam sHA/p54/p30 fusion			No	No	Yes	E75	66.6, 6	[224]
Modified vaccinia virus Ankara (MVA) p72+EP153R+CD2v	Georgia 2007/1		No	N/A	Yes	N/A	N/A	[221]
Adenoviral vectored p32+p54+p62+p72	Georgia 2007/1	ENABL and experimental adjuvant	Yes	N/A	Yes	N/A	N/A	[226]
Adenoviral vectored A151R+B119L+B602L, EP402RΔPRR+B438L+K205R/A104R	Georgia 2007/1	ENABL	Yes	N/A	Yes	N/A	N/A	[227]
Adenoviral vectored (Ad2) CP204L/E183L+EP402R+B646L/B602L	HLJ/18		Yes	N/A	Yes	China/GD/2019	100, 10	[230]
Adenoviral vectored (Ad5) cocktail with polycistronic constructs covering nearly 100% of ASFV proteome	Georgia 2007/1	None; Montamide USA-201^TM^; Biomize^®^	Yes	N/A	N/A	Georgia 2007/1	20, 5	[231]

^1^ “+”, simultaneous introduction; “/”, fusion. ^2^ sHA, soluble domain of CD2v. * N, the number of pigs vaccinated and then exposed to live ASFV. N/A, Not applicable.

## Data Availability

No new datasets were created.
